# Identification of Phosphoproteins as Possible Differentiation Markers in All-*Trans*-Retinoic Acid-Treated Neuroblastoma Cells

**DOI:** 10.1371/journal.pone.0018254

**Published:** 2011-05-05

**Authors:** Giorgia Mandili, Cristina Marini, Franco Carta, Cristina Zanini, Mauro Prato, Amina Khadjavi, Franco Turrini, Giuliana Giribaldi

**Affiliations:** 1 Dipartimento di Genetica, Biologia e Biochimica, Università di Torino, Torino, Italy; 2 Nurex S.R.L., Sassari, Italy; University of South Florida College of Medicine, United States of America

## Abstract

**Background:**

Neuroblastic tumors account for 9–10% of pediatric tumors and neuroblastoma (NB) is the first cause of death in pre-school age children. NB is classified in four stages, depending on the extent of spreading. A fifth type of NB, so-called stage 4S (S for special), includes patients with metastatic tumors but with an overall survival that approximates 75% at five years. In most of these cases, the tumor regresses spontaneously and regression is probably associated with delayed neuroblast cell differentiation.

**Methodology/Principal Findings:**

In order to identify new early markers to follow and predict this process for diagnostic and therapeutics intents, we mimicked the differentiation process treating NB cell line SJ-NK-P with all-*trans*-retinoic acid (ATRA) at different times; therefore the cell proteomic pattern by mass spectrometry and the phosphoproteomic pattern by a 2-DE approach coupled with anti-phosphoserine and anti-phosphotyrosine western blotting were studied.

**Conclusions/Significance:**

Proteomic analysis identified only two proteins whose expression was significantly different in treated cells versus control cells: nucleoside diphosphate kinase A (NDKA) and reticulocalbin-1 (RCN1), which were both downregulated after 9 days of ATRA treatment. However, phosphoproteomic analysis identified 8 proteins that were differentially serine-phosphorylated and 3 that were differentially tyrosine-phosphorylated after ATRA treatment. All proteins were significantly regulated (at least 0.5-fold down-regulated). Our results suggest that differentially phosphorylated proteins could be considered as more promising markers of differentiation for NB than differentially expressed proteins.

## Introduction

Neuroblastic tumors account for 9–10% of pediatric tumors and neuroblastoma (NB) is the most frequent extra-cranial solid tumor and the first cause of death in pre-school age children [1]. NB is heterogenous in nature and mirrors the variety of cells that originate from the embryonic neural crest. These cells are capable of differentiating into an assortment of cells, including peripheral neurons and melanocytes [2], [3], [4], [5], [6]. NB is a tumor composed of undifferentiated or poorly differentiated neuroblasts. The arrested differentiation of neuroblasts is apparently an early event in the pathogenesis of NB [7]. According to the International Neuroblastoma Staging System, NB is classified into 4 stages: 1, 2A–B, 3 and 4, depending on the extent of spreading. Each stage is characterized by different overall survival [1]. Prognostic factors for NB are available and widely applied in pediatric oncology. Factors currently used for risk stratification are histology (according to the International Neuroblastoma Pathology Classification [INPC] and the mitosis karyorrhexis index), proto-oncogene MYCN amplification, deletion of the short arm of chromosome 1 and ploidy (cellular deoxyribonucleic acid [DNA] content) [8]. However, markers that identify areas of cellular differentiation/maturation on histological sections and with more sensitivity or precocity than morphology alone might provide additional factors and help to stratify patients according to risk groups. Like other tumors, NB can regress spontaneously and occurs at a higher frequency in NB than in any other type of cancer. HNK-1, Trk-A, H-Ras, GRP78, GRP75 and calreticulin, are biologic factors already identified that predicted a favorable outcome for NB patients associated with the differentiation or regression of NB cells at early clinical stages [9], [10]. Spontaneous regression is most frequently observed in a particular subset of disseminated MYCN single-copy NB [non-amplified (NA)], termed stage 4S (stage 4S-NA, S for special) [11]. This category includes patients up to one year of age, showing metastatic tumors at onset that infiltrate skin, liver and/or bone marrow, but not bone [1], [12]. Stage 4S NB is one of the major puzzles for oncologists. Patients with disseminated tumors usually have very few chances to survive but stage 4S patients have an overall survival rate that approximates 75% at five years. In most of these cases, the tumor regresses spontaneously without any treatment or following supportive treatment only. Regression of this tumor is probably associated with differentiation and/or activation of programmed cell death [1]. Neuronal differentiation can be induced in neuronal precursor cells, including a wide number of NB cell lines, by exposure to all-*trans*-retinoic acid (ATRA), a retinoic acid isoform compound commonly used for *in vitro* NB differentiation models [5], [13], [14]. Specifically, exposure of cells to physiological concentrations of ATRA: (i) increases the number of cells bearing neuritic processes and the length of these processes; (ii) generally inhibits cell proliferation. ATRA exerts its effects on gene transcription by binding to nuclear retinoic acid receptors (RARs) [5]. Although non-genomic actions of retinoids have been proposed, they have not been well investigated. In some cases, ATRA has been shown to rapidly activate protein kinases including extracellular-regulated kinase 1/2 [15], [16], [17], [18], [19] c-Jun N-terminal kinase [18], [20], and phosphatidylinositol 3-kinase [18], [21]. Protein kinase activation has been proposed to play a role in modulating neurite outgrowth [5] but protein substrates have not been comprehensively studied. In other cases retinoic acid has been shown to activate protein phosphatases, such as mitogen-activated protein kinase phosphatase I [22], protein phosphatase 2A and protein phosphatase 2B [23].

In this paper, we performed comparative proteomic and phosphoproteomic analysis using a 2-DE approach coupled with anti-phosphoserine and anti-phosphotyrosine western blotting. Our model was the human SJ-NK-P NB cell line, treated or not with ATRA for different times, to look for new potential differentiation markers.

## Results

### Effects of ATRA on NB cell morphology

**Figure 1:**
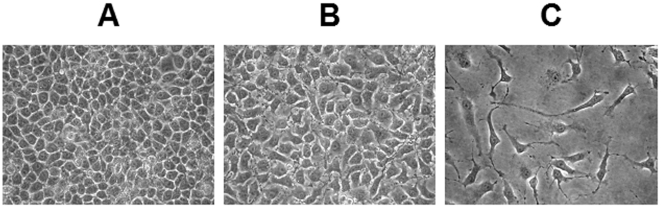
Phase contrast microscopy of NB cell line treated or not with ATRA. (**A**) SJ-N-KP control cells. (**B**) SJ-N-KP cells treated with 10 µM ATRA for 24 hours. (**C**) SJ-N-KP cells treated with 10 µM ATRA for 9 days. Magnification: 40×.

SJ-N-KP cells were treated with 10 µM ATRA for 24 hours and 9 days. Figure 1 shows that after 24 hours few but consistent differences in cell morphology were already appreciable in treated cells, which started to elongate in comparison with control cells. After 9 days, ATRA-treated cells exhibited an extensive network of neurite process highlighting an evolution towards cell differentiation. Therefore ATRA induced differentiation in the SJ-N-KP NB cell line as expected.

### Effect of ATRA on protein expression

**Figure 2:**
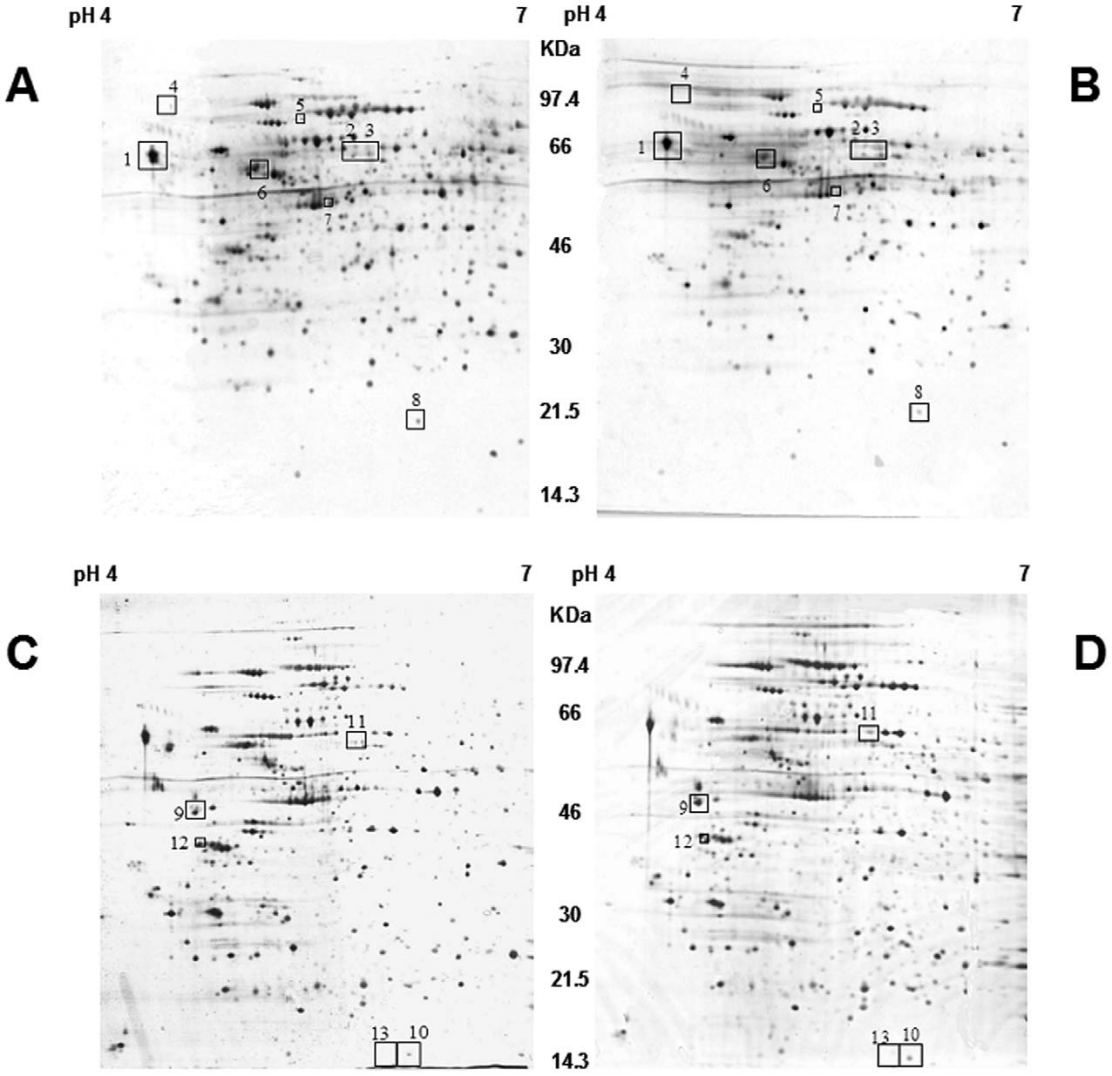
2-DE analysis of NB cells treated or not with ATRA. Representative image from three independent experiments of coomassie-stained 2-DE patterns of NB cell line SJ-N-KP: (**A**) 24 h control cells; (**B**) 24 h ATRA treatment; (**C**) 9 days control cells; (**D**) 9 days ATRA treatment. Proteins were separated using 17-cm, pH-4–7 strips followed by SDSPAGE on 10%, 18×20-cm gels. Proteins showing apparent differential expression were identified by progressive numbers on the *black squares*. Corresponding identifications are reported in Table 1 and 2.

In order to find new differentiation markers for NB, cell differentiation was mimicked by treatment with 10 µM ATRA. Comparative analysis of protein expression by 2-DE and mass spectrometry was then performed in SJ-N-KP cells treated or not for 24 hours or 9 days. Figure 2 shows representative 2-DE gel images of control cells (Figure 2A, 24 h and 2C, 9 days) and ATRA-treated cells (Fig. 2B, 24 h and 2D, 9 days) stained by comassie. Protein separation was very efficient, allowing the study of more than 500 protein spots. Tables 1 and 2 report the mass spectrometry identifications of all differentially expressed proteins. Seven proteins were differentially expressed (defined as at least 1.5-fold upregulated or 0.5-fold downregulated) in samples treated with ATRA for 24 hours (Table 1) and 5 proteins were differentially expressed in samples treated for 9 days (Table 2). However, the differential expression was statistically significant only for two proteins and only at the longest treatment time: nucleoside diphosphate kinase A (NDKA) (p<0.05) and reticulocalbin-1 (RCN1) (p<0.01), which were significantly downregulated after 9 days of ATRA treatment.

**Table 1:** Proteins differentially expressed in neuroblastoma cell line following 24 h ATRA treatment as identified by MALDI-Tof MS.

Spot number^a^	Identified protein	Accession number	MW (Da)^b^	pI^c^	Mascot Score	Matching Peptide/Searched^d^	Coverage (%)^e^	Treated/Untreated Ratio ± SD^f^
1	Calreticulin precursor	P27797	48283	4.29	121	11/25	27	3.1±4.5
2	Vimentin	P08670	53676	5.06	137	13/25	36	2.2±1.7
3	Vimentin	P08670	53676	5.06	88	11/25	24	21.2±35.1
4	78 kDa glucose-regulated protein precursor	P11021	72402	5.07	77	9/25	19	1.9±1.6
5	Ubiquilin-2	Q9UHD9	65655	5.15	74	9/25	17	4±5.3
6	Protein disulfide-isomerase precursor	P07237	57480	4.76	127	12/25	30	3.9±1.8
7	ATP synthase subunit beta, mitochondrial precursor	P06576	56525	5.26	163	16/25	32	2.4±1.2
8	Glutathione S-transferase P	P09211	23569	5.43	56	5/25	27	3.1±2.1

a Spot number were defined according to spot positions in 2-DE in Fig. 2.

b MW, molecular weight.

c pI, isoelectric point.

d Number of matched mass values on number of total mass values searched.

e The sequence coverage, which is calculated as the percentage of identified sequence to the complete sequence of the matched protein.

f Ratio between level of expression in treated and untreated cells. Standard deviation is indicated. For the significance two-sided Student's *t* test was used (* p<0.05, ** p<0.01).

**Table 2:** Proteins differentially expressed in neuroblastoma cell line following 9 days ATRA treatment as identified by MALDI-Tof MS.

Spot number^a^	Identified protein	Accession number	MW (Da)^b^	pI^c^	Mascot Score	Matching Peptide/Searched^d^	Coverage (%)^e^	Treated/Untreated Ratio ± SD^f^
9	Reticulocalbin-1 precursor	Q15293	38866	4.86	61	6/25	20	0.4±0.1**
10	Nucleoside diphosphate kinase A	P15531	17309	5.83	76	6/25	40	0.3±0.3*
11	Vimentin	P08670	53676	5.06	95	10/25	26	3.9±4.4
12	Nucleophosmin	Q96EA5	32726	4.64	60	5/25	25	3.9±2.9
13	Deoxyuridine 5′-triphosphate nucleotidohydrolase, mitochondrial precursor	P33316	26975	9.65	80	6/25	34	1.9±1.8

a Spot number were defined according to spot positions in 2-DE in Fig. 2.

b MW, molecular weight.

c pI, isoelectric point.

d Number of matched mass values on number of total mass values searched.

e The sequence coverage, which is calculated as the percentage of identified sequence to the complete sequence of the matched protein.

f Ratio between level of expression in treated and untreated cells. Standard deviation is indicated. For the significance two-sided Student's *t* test was used (* p<0.05, ** p<0.01).

### Effect of ATRA on protein phosphorylation

**Figure 3:**
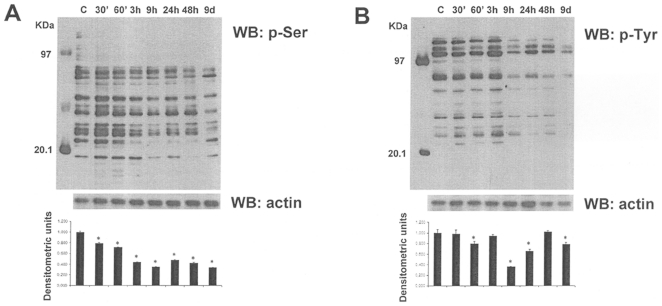
1-DE western blot analysis of NB cells treated or not with ATRA. Representative western blot (of three independent experiments) with anti-phosphoserine (**A**) and anti-phosphotyrosine (**B**) antibodies. SJ-N-KP cells were treated with ATRA 10 µM for 30, 60 minutes, 3, 9, 24, 48 hours and 9 days. Control cells (c) were not treated. 15 µg of proteins were analysed on 10% SDS-polyacrylamide gel. Western blot analysis with anti-actin antibodies was performed on the same membranes and used for data normalization. Data (means ± SD) expressed as densitometric units are shown in the lower corner of panels **A** and **B**. Significance of the differences between controls and ATRA treated cells: *p*≤0.05.

**Figure 4:**
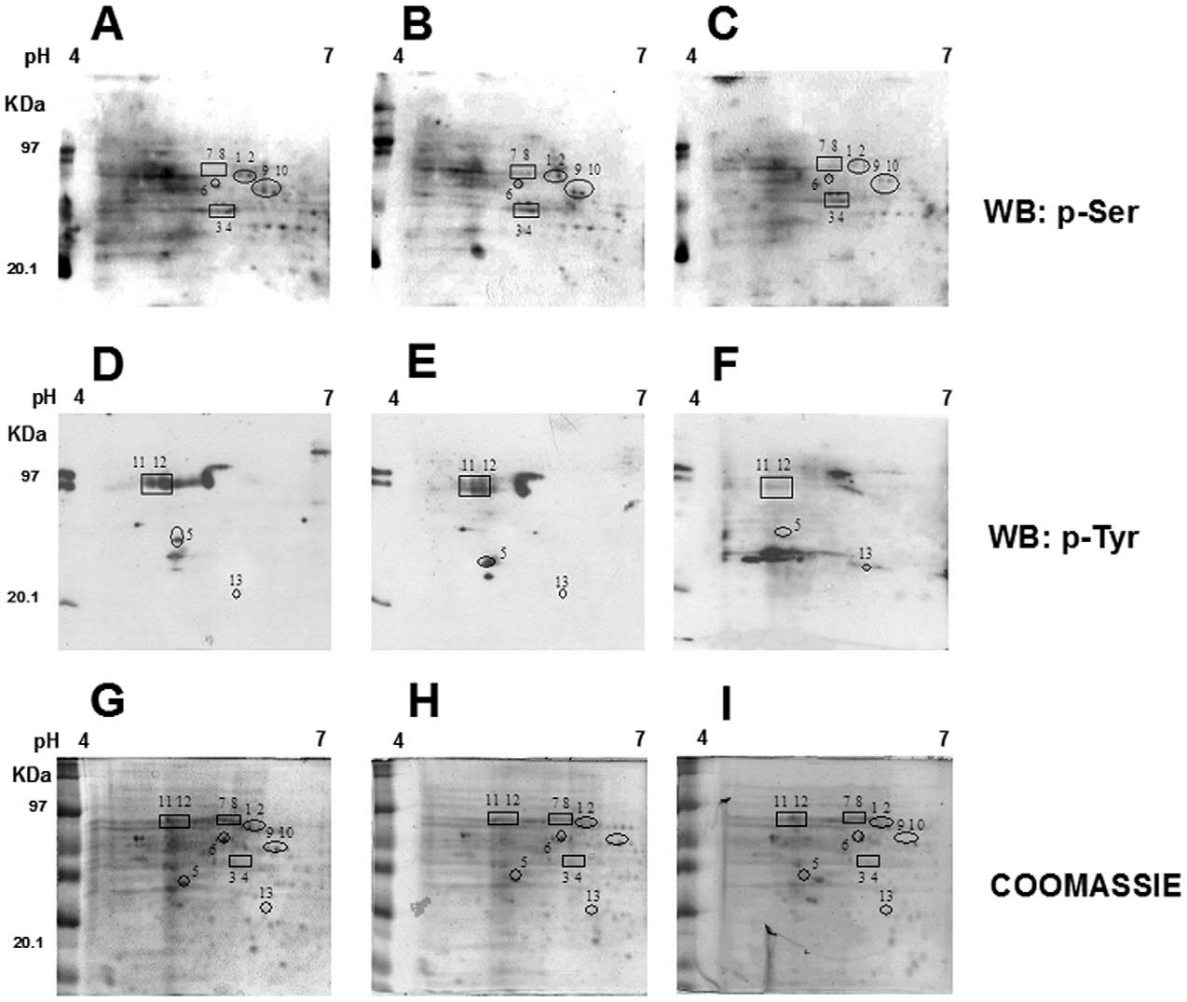
Phosphoproteome analysis of NB cell line treated or not with ATRA. Representative 2-DE western blot (of three independent experiments) with anti-phosphoserine (**A**, **B**, **C**) and anti-phosphotyrosine (**D**, **E**, **F**) antibodies are shown. Proteins were separated using 7-cm, pH-4–7 strips followed by SDSPAGE on 10%, 8×7-cm gels. (**A**, **D**) SJ-N-KP control cells. (**B**, **E**) SJ-N-KP cells treated with 10 µM ATRA for 30′. (**C**, **F**) SJ-N-KP cells treated with 10 µM ATRA for 60′. The *black squares and circles* give the position of identified protein spots that are differentially phosphorylated in ATRA treated cells in comparison to control cells. The spot numbers correspond to the spot numbers listed in Table 3. Panels **G**, **H** and **I** show the representative (of three independent experiments) coomassie-stained 2-DE patterns of NB cell line SJ-N-KP not treated (**G**) and treated with ATRA for 30 minutes (**H**) and for 60 minutes (**I**). 2-DE was performed as described above.

**Figure 5:**
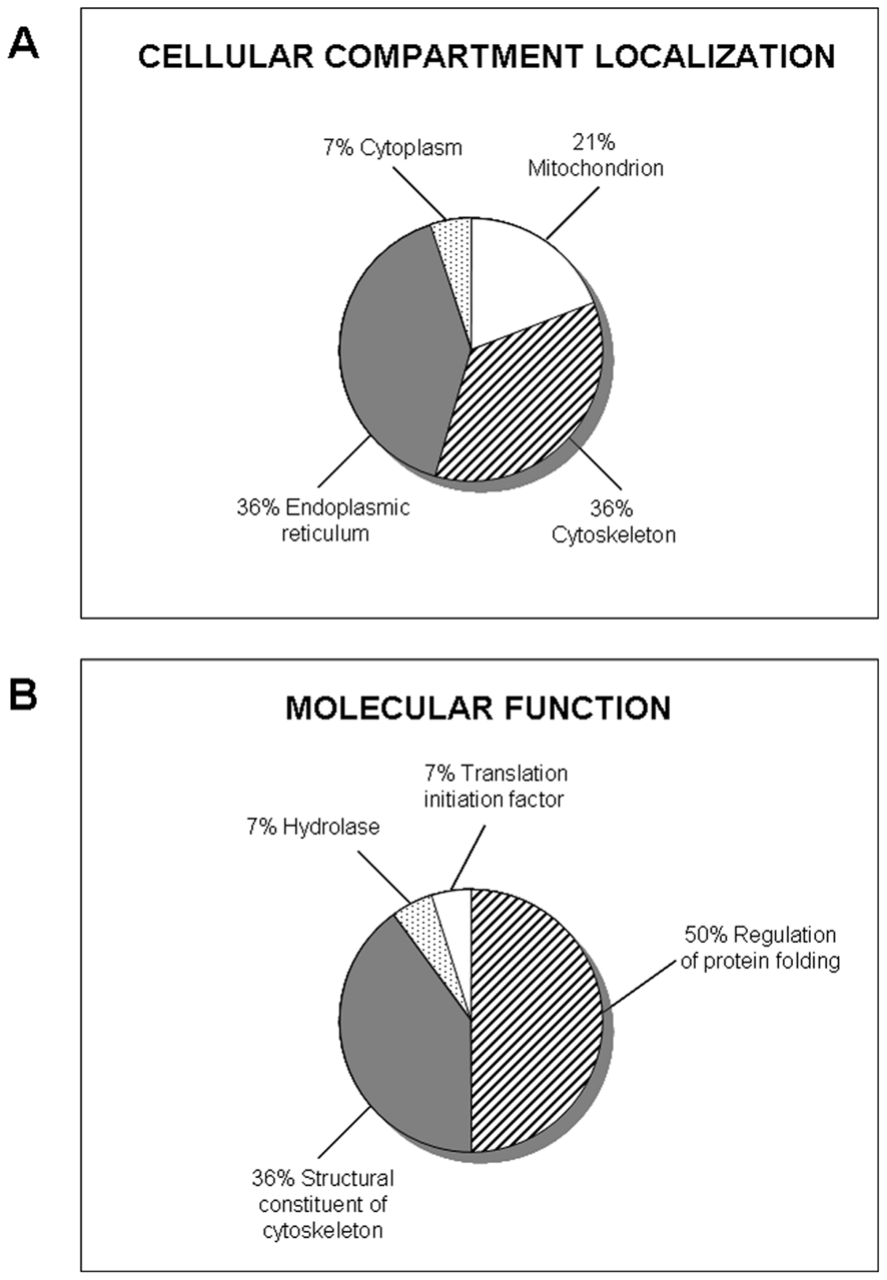
Distribution of identified spots corresponding to proteins differentially phosphorylated following ATRA treatment. Distribution is shown according to proteins cellular compartment localization and their molecular function.

Since the phosphoproteome changes during cancer development and phosphoproteins may constitute useful markers for the early individuation of NB differentiation process, the effects of ATRA on serine- and tyrosine- phosphorylation patterns in NB cells were investigated. Firstly, we performed 1-DE analysis followed by western blotting with anti-phosphoserine and anti-phosphotyrosine antibodies in SJ-N-KP control cells and cells treated with 10 µM ATRA for 30 min, 60 min, 3 h, 9 h, 24 h, 48 h or 9 days. Figure 3 shows that even though serine- and tyrosine-phosphorylated proteins showed the same pattern in ATRA-treated and untreated cells, protein phosphorylation levels were significantly different in ATRA-treated cells at different times in comparison with control cells as shown by densitometric analysis of phosphoserine (panel A) and phosphotyrosine (panel B) western blotting. To better understand the effects of ATRA on protein phosphorylation, 2-DE analysis followed by western blotting with anti-phosphoserine and anti-phosphotyrosine antibodies was performed in cells treated or not with ATRA for 30 min and 60 min. These two time periods were selected in order to identify the early changes induced by ATRA. Figure 4 shows the representative phosphorylation pattern in control cells (Fig. 4A and 4D), in cells treated with ATRA for 30 minutes (Fig. 4B and 4E) and in cells treated with ATRA for 60 minutes (Fig. 4C and 4F). Proteins found to be differentially phosphorylated and further identified are evidenced. In agreement with a greater rate of serine phosphorylation with respect to tyrosine phosphorylation, we observed more spots in anti-phosphoserine (upper panels) than in anti-phosphotyrosine (lower panels) western blots (even though more proteins were loaded for anti-phosphotyrosine western blots than for anti-phosphoserine blots). Table 3 reports the differentially phosphorylated proteins identified by mass spectrometry. We identified 1 protein that was differentially serine-phosphorylated after ATRA treatment at 30 minutes, and 7 proteins (from 8 spots) at 30 or 60 minutes. We also identified 1 protein (from 2 spots) that was differentially tyrosine phosphorylated at 30 and 60 minutes, and 2 proteins at 60 minutes. All differences were statistically significant (minimun cut-off: 0.5-fold down-regulation). It is of note that after 30 and 60 minutes of ATRA treatment we did not observe any changes in protein expression (Fig. 4 panels G, H, I). The differentially phosphorylated proteins identified were classified into different groups depending on their cellular compartment localization and molecular function (Fig. 5 A and B).

**Table 3:** Proteins differentially phosphorylated in neuroblastoma cell line following ATRA treatment as identified by MALDI-Tof MS.

Spot number^a^	Identified protein	Accession number	MW (Da)^b^	pI^c^	Mascot Score	Matching Peptide/Searched^d^	Coverage (%)^e^	p-Ser 30′: Treated/Untreated Ratio ± SD^f^	p-Ser 60′: Treated/Untreated Ratio ± SD^f^	p-Tyr 30′: Treated/Untreated Ratio ± SD^f^	p-Tyr 60′: Treated/Untreated Ratio ± SD^f^
1	Stress-70 protein, mitochondrial precursor	P38646	73920	5.87	85	11/25	15	0.3±0.3**	0.5±0.4		
2	ATP-dependent Clp protease ATP-binding subunit ClpX-like, mitochondrial precursor	O76031	69922	7.51	57	6/25	14	0.6±0.9	0.4±0.3*		
3	Actin, cytoplasmic 1	P60709	42052	5.29	124	11/25	36	0.5±0.4	0.4±0.2*		
	Actin, cytoplasmic 2	P63261	42108	5.31	124	11/25	36				
4	Actin, cytoplasmic 1	P60709	42052	5.29	96	8/25	26	0.2±0.4*			
	Actin, cytoplasmic 2	P63261	42108	5.31	96	8/25	26				
5	Actin, cytoplasmic 1	P60709	42052	5.29	77	8/25	26				0.1±0.1**
	Actin, cytoplasmic 2	P63261	42108	5.31	77	8/25	26				
6	ATP synthase subunit beta, mitochondrial precursor	P06576	56525	5.26	159	15/25	41	0.3±0.3*	0.4±0.8		
	Protein disulfide-isomerase A6 precursor	Q15084	48490	4.95	58	6/25	26				
7	Vimentin	P08670	53676	5.06	253	20/25	54	0.3±0.4	0.5±0.5*		
8	Vimentin	P08670	53676	5.06	58	7/25	22	0.1±0.2	0.2±0.4*		
9	Protein disulfide-isomerase A3 precursor	P30101	57146	5.98	151	15/25	34	24.9±42.4	0.2±0.1**		
10	Protein disulfide-isomerase A3 precursor	P30101	57146	5.98	138	12/25	26	1.3±0.7	0.4±0.3*		
11	78 kDa glucose-regulated protein precursor	P11021	72402	5.07	174	16/25	28			0.1±0.2**	1±1.4
12	78 kDa glucose-regulated protein precursor	P11021	72402	5.07	189	17/25	30			0.3±0.5**	0.1±0.1**
13	Eukaryotic translation initiation factor 3 subunit 2	Q13347	36878	5.38	157	13/25	45				0.2±0.2*

a Spot number were defined according to spot positions in 2-DE in Fig. 4.

b MW, molecular weight.

c pI, isoelectric point.

d Number of matched mass values on number of total mass values searched.

e The sequence coverage, which is calculated as the percentage of identified sequence to the complete sequence of the matched protein.

f Ratio between level of phosphorylation in treated and untreated cells. Standard deviation is indicated. For the significance two-sided Student's *t* test was used (* p<0.05, ** p<0.01).

## Discussion

NB shows the highest rate of spontaneous regression of any human tumor, mainly due to differentiation and maturation of these highly malignant cells into neurons [24]. It is of interest to study the molecular pathways driving spontaneous regression in NB to unravel the molecular basis of NB development, to look for new potential differentiation markers and to determine the differentiation therapies that can be used to treat patients. Proteome analysis is useful to identify differentially expressed proteins as possible new biomarkers, and the analysis of protein phosphorylation allows the study of the early events in the differentiation process, being thus an important goal in the identification of new early expressed biomarkers. Phosphorylation events in cellular signaling cascades triggered by a variety of cellular stimuli modulate protein function, leading to diverse cellular outcomes including cell division, growth, differentiation and death. Abnormal regulation of protein phosphorylation due to mutation or overexpression of signaling proteins often results in various disease states [25]. Phosphoproteome changes occurring during cancer development and phosphoproteins could constitute biomarkers useful for cancer diagnostics and therapeutics [26]. It is well-known that the phosphotyrosine proteome in breast and liver cancer is indeed distinct [26]. In addition, Khadjavi et al. recently demonstrated that the protein tyrosine phosphorylation pattern is profoundly different in bladder cancer tissue biopsies in comparison with the surrounding healthy tissues and that phosphorylated proteins may represent a new valuable class of bladder cancer urinary markers (Khadjavi et al., *in press*). In this report, the phosphorylation status of ATRA-treated NB cells was studied using a 2-DE approach coupled with anti-phosphoserine and anti-phosphotyrosine western blotting and by mass spectrometry. Recent advances in mass spectrometry have clearly revolutionized the studies of phosphoprotein biochemistry, but gel electrophoresis-based proteomics remains one of the most appropriate methods for analyzing protein phosphorylation [27]. The present study identified several proteins with strikingly different expression or phosphorylation patterns in ATRA-treated NB cells with respect to control cells. However, more intense and evident changes in ATRA-treated cells were found in their protein phosphorylation pattern: 10 proteins were differentially phosphorylated already after 30 or 60 min treatment. In contrast, just 2 proteins were differentially expressed 9 days after treatment). The lower number of statistically significant differences among proteins expressed after 24 h and 9 days of treatment can be ascribed to the observed variability between different cell preparations [28] related to the tendency of NB cells to undergo spontaneous differentiation in vitro [29]. The 2-DE phosphorylation pattern showed more phosphoserine than phosphotyrosine residues according to the major presence of aminoacid residues modified by phosphorylation in serine (P-Ser≅90%) than in tyrosine (P-Tyr≅0.05%) [30]. The response observed to ATRA treatment is not an on/off-like response, but a variation in phosphorylation levels. This is in accordance with the fact that the biologically significant aspect of protein phosphorylation is especially related to the quantitative variation of the state of phosphorylation on a given protein or groups of proteins [27]. Curiously, here almost all proteins identified that changed their phosphorylation levels in a statistically significant way tended to decrease their phosphorylation levels following ATRA treatment. This observation leads us to postulate the involvement of protein phosphatases in NB differentiation. At present, few data supporting this hypothesis are available. Den Hertog et al. demonstrated that receptor protein tyrosine phosphatase α expression is enhanced during neuronal differentiation of embryonal carcinoma cells and N1E-115 NB cells [31]. Additionally, Haque et al. reported the upregulation of the activities of both protein phosphatase 1 and protein phosphatase 2A in NB cells after differentiation [32]. Major studies are undoubtedly required to confirm this intriguing hypothesis. Thus, the evidence showed in the present work might be placed within this context and further investigation on the role of phosphatases in NB differentiation is warranted. On the other hand, a large body of data reports that ATRA treatment promotes protein phosphorylation through the activation of several protein kinases [15], [16], [17], [18], [19], [20], [21]. However, in many cases the biological meaning of these phosphorylative events needs to be elucidated.

In the present work, among the proteins with decreased phosphorylation levels at the beginning of differentiation program induced by ATRA, we found 3 mitochondrial proteins: stress-70 protein, ATP synthase and ATP-dependent Clp protease. It has been already demonstrated that the mitochondrial phosphoproteome comprises proteins belonging to different functional groups: metabolism, apoptosis, cell cycle, cell maintenance, transcription/translation, structural, chaperones and stress response [33]. As shown by Pagliarini et al., reversible phosphorylation is becoming an important means of regulating mitochondrial functions [34]. In this case, decreased stress-70 protein serine-phosphorylation levels can be related to the observed inhibition of human NB growth induced by ATRA [5], [14], in agreement with evidence showing that, on the contrary, high levels of stress-70 protein serine-phosphorylated accelerate centrosome duplication and cellular mitosis [35]. The role of phosphorylation of the ATP synthase β subunit has been elucidated, indicating that the phosphorylation event precedes the recruitment of mitochondrial 14-3-3 proteins with consequent inhibition of ATP synthesis [36]. Some data are apparently in contrast with the present evidence showing a decrease of phosphorylation levels of the β subunit of the ATP synthase following ATRA treatment. For example, increased phosphorylation of mitochondrial ATP synthase beta-chain in apoptotic and in terminally differentiated HL-60 cells has been demonstrated [37]. Of note, in the cited paper, ATRA treatment was prolonged for 120 hours. ATP-dependent Clp protease was already seen to be phosphorylated on serine in a leukemia cell line [38] but no data correlating protein phosphorylation and differentiation are currently available.

Among the differentially phosphorylated proteins identified here, a second group belonged to ER proteins: serine-phosphorylated protein disulfide-isomerase A6, serine-phosphorylated protein disulfide-isomerase A3 and tyrosine-phosphorylated 78 kDa glucose-regulated protein. As described below, all these proteins are involved in the ER stress response, which is notably induced by retinoic acid [39], [40]. Protein disulfide-isomerase A6 phosphorylation on serine is well-documented [41], [42], [43], [44] and there is evidence that this protein is serine-phosphorylated in G1 and M phases [44]; thus a decrease of phosphorylation of this protein fits well with data showing the inhibition of proliferation induced by ATRA [5], [14]. Phosphorylation of disulfide-isomerase A3 is induced by a variety of stimuli such as leptin in the liver, angiotensin II in smooth muscle cells and gamma knife surgery in the brain; it is thought that the subcellular localization of protein disulfide-isomerase A3 is regulated by the phosphorylation of the tyrosine and serine residues [45]; however no data correlating protein phosphorylation and differentiation are available. Finally, it is well known that 78 kDa glucose-regulated protein can be tyrosine phosphorylated; it is a Src substrate [46] which must be tyrosine-phosphosylation for activation of PAK, a kinase important in mobility of malignant cells and proliferation [47]. Therefore the observed dephosphorylation of the 78 kDa glucose-regulated protein could be linked to data showing the inhibition of proliferation induced by ATRA [5], [14]. Another category of proteins identified as differentially phosphorylated are the cytoskeleton proteins actin and vimentin. Both are known to be phosphorylated [48], [49], [50], [51], [52] in conjunction with cytoskeleton reorganization, which may conceivably occur during differentiation [53]. In particular, serine-phosphorylation of vimentin by Rho-associated kinase is involved in the agonist-induced neurite retraction of neuronal cells in NB [50]; these data are in accordance with our observation of a a diminution of phosphorylation levels following differentiation in NB leading to neurite spreading. Moreover, the expression of vimentin also seems to be involved in NB differentiation induced by ATRA, but the data are not exhaustive [54], [55]. Finally, eukaryotic translational initiation factor 3, a cytoplasmic protein involved in the initiation of protein synthesis, was seen to be phosphorylated on tyrosine residues in fibroblast and lung cancer cell lines [56], [57]; no data are available correlating its phosphorylation and differentiation.

Protein localisation is certainly important, but it also interesting to classify the described proteins according to their functions. Among the proteins identified as being differentially phosphorylated in present work, many are involved in folding mechanisms: stress-70 protein, ATP-dependent Clp protease, protein disulfide-isomerase A6, protein disulfide-isomerase A3, 78 kDa glucose-regulated protein. This is compatible with the reorganization of cellular status that occurs during differentiation with probable concomitant synthesis of new proteins. On the other hand, other proteins are glucose-related: protein disulfide-isomerase A3, 78 kDa glucose-regulated protein and stress-70 protein. 78 kDa glucose-regulated protein is essential for the differentiation of NB cells [9]. Moreover, it is known that 78 kDa glucose-regulated protein is also influenced by ATRA, but the effect appears to be strictly connected to cell origin, as ATRA downregulates 78 kDa glucose-regulated protein expression in foetal bovine chondrocytes [58] but upregulates its expression in hepatocarcinoma cells [59]. Stress-70 protein and protein disulfide-isomerase A3 have already been correlated with ATRA and differentiation, being overexpressed in NB cells following ATRA treatment [9], [10].

In conclusion, protein phosphorylation status in NB cells could be an interesting potential biomarker of early differentiation. A future analysis of the phosphorylation status of proteins in NB tissues utilizing specific phosphorylated-protein antibodies could permit the validation of these proteins as candidates for application in the clinical practice. The use of markers of maturation could be very important to detect the onset of differentiation and to choose the most appropriate clinical treatment. Additionally, there is increasing interest in the application of differentiation inducers in the treatment of NB, possibly in order to mimick the differentiation process that occurs in stadium 4S-tumours, which can regress spontaneously [7]. Thus, a more exhaustive comprehension of the phenomena described in the present study can also help to better understand the differentiation mechanisms in forms of NB that do not regress spontaneously.

## Materials and Methods

### Chemicals and reagents

RPMI 1640, streptomycin, penicillin, ATRA, protease and phosphatase inhibitors, ammonium persulfate (APS), bromophenol blue, glycerol, N,N,N′,N′-tetramethylethylene-diamine (TEMED), sodium dodecyl sulfate (SDS), TRIZMA, urea, 3-[(3-cholamidopropyl) dimethylammonio]-1-propanesulphonate (CHAPS), dithiothreitol (DTT), iodoacetamide were purchased from Sigma-Aldrich (St. Louis, MO, USA). DC Protein assay kit, acrylamide, agarose, ready-made immobilized pH gradient (IPG) strip (7- and 17-cm IPG strips, pH 4–7), enhanced chemiluminescence kit were purchased from Bio-Rad (Hercules, CA, USA). Ampholine pH 3.5–10 and 5–8 were obtained from GE Healthcare (MI, ITALY). Fetal bovine serum USA Origin was purchased from EuroClone (Paignton, UK). PVDF membrane Immobilon-P was obtained from Millipore (MI, Italy). Antibodies anti-phosphoserine PSR-45, anti-phosphotyrosine PY-20 and anti-goat IgG horseradish-peroxidase-labeled were purchased from Sigma, anti-phosphotyrosine PY99 and anti-actin from Santa Cruz Biotechnology (Santa Cruz, CA, USA) and anti-mouse IgG horseradish-peroxidas e-labeled from GE Healthcare Bio-Sciences.

### Cell culture and ATRA treatment

Established NB human cell line SJ-N-KP [60] was cultured at 37°C, 5% CO_2_ in RPMI 1640 supplemented with 10% heat inactivated foetal bovine serum USA Origin, 100 µg/mL streptomycin and 100 U/mL penicillin. Cells were cultured in the presence or not of 10 µM [61], [62] ATRA at different times: 24 hours and 9 days for morphological analysis; 30, 60 minutes, 24 hours and 9 days for protein expression analysis experiments; 30, 60 minutes, 3, 9, 24, 48 hours or 9 days for protein phosphorylation analysis experiments. The medium was changed every two days.

### Morphological analysis

Subconfluent cells were treated or not with 10 µM ATRA, as previously described. Cell morphology was investigated with phase contrast microscopy (microscope Axiovert 35, Zeiss) and photographed with magnification 40× with a digital camera (Coolpix N25, Nikon).

### Sample preparation for protein expression and phosphorylation analysis experiments

One confluent dish (150×25 mm) was washed two times in PBS harvested with a scraper and suspended in 0.5 ml of a solution containing 8 M urea, 2% w/v CHAPS, 40 mM Trizma, protease and phosphatase inhibitors. The sample was incubated O.N. at 4°C and spun down at 13,800 g for 10 min at 4°C. The clear supernatant was removed, quantified with DC Protein assay kit and stored at −20°C until analysis.

### Mono-dimensional electrophoresis (1-DE)

After adding of Laemmli buffer [63], samples were boiled for 5 min and 60 mM final concentration DTT, was added. 15 µg of each protein sample were analysed on 10% SDS-polyacrylamide gel on the Mini Protean system (Bio-Rad).

### Two-dimensional gel electrophoresis (2-DE)

2-DE was performed using ready-made IPG strip (17- and 7-cm IPG strips, pH 4–7). Each sample (150 µg of protein for large analytical gels and 2 mg for large preparative gels, 100 µg for anti-phosphoserine western blotting and 300 µg for anti-phosphotyrosine western blotting and preparative gels) was applied onto an IPG gel by in-gel rehydration for 20 h, adding DTT 1% w/v, final concentration and Ampholine pH 3.5–10, 2% v/v, final concentration. Isoelectric focusing was carried out in a Protean IEF cell apparatus (Bio-Rad). Briefly, focusing for 7-cm IPG strips was started at 250 V, and the voltage was progressively increased to 4000 V until a maximum of 25000 V-h was reached; for 17-cm IPG strips, the voltage was increased to 9000 V until a maximum of 60000 V-h was reached. Focusing was performed at 18°C with a limit of 50 µA per strip. Subsequently the IPG strips were equilibrated under continuous shaking for 15 min in equilibration buffer 1 (6 M urea, 2% w/v SDS, 0.05 M Tris–HCl pH 8.8, 20% v/v glycerol, 1% w/v DTT) and for 12 min in equilibration buffer 2 (6 M urea, 2% w/v SDS, 0.05 M Tris–HCl pH 8.8, 20% v/v glycerol, 2.5% w/v iodoacetamide). For small format second dimension, 8×7×0.1 cm 10% acrylamide gels were run on the Mini Protean system (Bio-Rad). For large format second dimension, 18×20×0.1 cm 10% acrylamide gels were run on the Bio-Rad XI cell. Electrophoresis was performed for 30 min at 50 V and then was continued at 100 V until the Bromophenol blue front reached the lower limit of the gel. Analytical gels were stained with silver staining as described [64] and preparative gels with colloidal Coomassie (18% v/v ethanol, 15% w/v ammonium sulfate, 2% v/v phosphoric acid, 0.2% w/v Coomassie G-250) for 48 h and destained with water.

### Western blotting

Small format gels were transferred to a PVDF membrane. Membranes were blocked with 3% w/v BSA in PBST (PBS supplemented by polyoxyethylenesorbitan monolaurate [Tween 20] 0.1% v/v) for 1 h and then membranes were washed three times with PBST and probed with a 1∶400 dilution of anti-phosphoserine PSR-45 or 1∶400 dilution of two mixed 1∶1 anti-phosphotyrosine (PY20 and PY99) O.N. at 4°C. Membranes were washed six times with PBST, incubated for 1 hour with a 1∶10000 dilution of anti-mouse IgG horseradish-peroxidase-labeled antibody and immunoreactivity was detected with an enhanced chemiluminescence kit. Afterwards membranes with 1-DE analysis were stripped (2 hours washing with glycine 20 mM pH 2.2, Tween 1% v/v, SDS 0.1% w/v) and probed with 1∶100 dilution of anti-actin antibodies for normalization of the data.

### Image analysis

1-DE densitometric analysis was performed using ImageJ software (version 1.44, free). 2-DE image analysis was performed using PD-Quest software (version 7.2, Bio-Rad) according to the manufacturer's instructions. Normalization of each individual spot was performed according to the total quantity of the valid spots in each gel, after subtraction of the background values. The spot volume was used as the analysis parameter to quantify protein expression or phosphorylation.

### Protein identification by mass spectrometry and database search

Coomassie G-stained spots were excised from 2-DE preparative gels; destaining and in-gel enzymatic digestion were performed as previously described [65]. Briefly each spot was destained with 100 µl of 50% v/v acetonitrile in 5 mM ammonium bicarbonate and dried with 100 µl of acetonitrile. Each dried gel piece was rehydrated for 40 min at 4°C in 10 µl of a digestion buffer containing 5 mM ammonium bicarbonate, and 10 ng/µl of trypsin. Digestion was allowed to proceed overnight at 37°C and the peptides mixtures were stored at 4°C until assayed. All digests were analyzed by MALDI-TOF (TofSpec SE, MicroMass) equipped with a delayed extraction unit. Peptides solution was prepared with equal volumes of saturated α-cyano-4-hydroxycinnamic acid solution in 40% v/v acetonitrile-0.1% v/v trifluoroacetic acid. The MALDI-TOF was calibrated with a mix of PEG (PEG 1000, 2000 and 3000 with the ratio 1∶1∶2) and mass spectra were acquired in the positive-ion mode. Peak lists were generated with ProteinLynx Data Preparation (ProteinLynx Global Server 2.2.5) using the following parameters: external calibration with lock mass using mass 2465.1989 Da of ACTH, background subtract type adaptive combining all scans, performing deisotoping with a threshold of 1%. The 25 most intense masses were used for database searches against the SWISSPROT database (release 2011-01 of 11-Jan-11) using the free search program MASCOT 2.3.02 (http://www.matrixscience.com). The following parameters were used in the searches: taxa *Homo sapiens*, trypsin digest, one missed cleavage by trypsin, carbamidomethylation of cysteine as fixed modification, methionine oxidation and serine or tyrosine phosphorylation (only for differential phosphoproteomic analysis) as variable modifications and maximum error allowed 100 ppm. Were taken on to consideration only protein with a Mascot score >55.

### Statistical analysis

Data from densitometric analysis were used as values of proteins phosphorylation in 1-DE experiments. Data from image analysis were used as values of protein expression or phosphorylation in 2-DE experiments. To verify the significance of the variations of expression and phosphorylation of proteins in ATRA treated cells *versus* control cells, two-sided Student's *t* test was used. Experiments were performed in triplicate. Statistical significance was set at *p*-values≤0.05. In 2-DE experiments proteins were classified as differentially expressed or phosphorylated if ratio in spot intensity between treated cells and control cells was greater than 1.5-fold (protein overexpressed or overphosphorylated) or lower than 0.5-fold (protein underexpressed or underphosphorylated).
